# Pt/CNT Micro-Nanorobots Driven by Glucose Catalytic Decomposition

**DOI:** 10.34133/2021/9876064

**Published:** 2021-08-06

**Authors:** Hao Wang, Jiacheng Kan, Xin Zhang, Chenyi Gu, Zhan Yang

**Affiliations:** Robotics and Microsystems Center, College of Mechanical and Electric Engineering, Soochow University, China

## Abstract

Swimming micro-nanorobots have attracted researchers' interest in potential medical applications on target therapy, biosensor, drug carrier, and others. At present, the experimental setting of the swimming micro-nanorobots was mainly studied in pure water or H_2_O_2_ solution. This paper presents a micro-nanorobot that applied glucose in human body fluid as driving fuel. Based on the catalytic properties of the anode and cathode materials of the glucose fuel cell, platinum (Pt) and carbon nanotube (CNT) were selected as the anode and cathode materials, respectively, for the micro-nanorobot. The innovative design adopted the method of template electrochemical and chemical vapor deposition to manufacture the Pt/CNT micro-nanorobot structure. Both the scanning electron microscope (SEM) and transmission electron microscope (TEM) were employed to observe the morphology of the sample, and its elements were analyzed by energy-dispersive X-ray spectroscopy (EDX). Through a large number of experiments in a glucose solution and according to Stoker's law of viscous force and Newton's second law, we calculated the driving force of the fabricated micro-nanorobot. It was concluded that the structure of the Pt/CNT micro-nanorobot satisfied the required characteristics of both biocompatibility and motion.

## 1. Introduction

The driving technology and the biocompatibility problem are the essential challenges faced by the design and manufacture of micro-nanorobots for medical applications [1, 2]. Micro-nanorobots can have various driving methods, including electric field driving, magnetic field driving [3, 4], chemical energy driving [5, 6], and so on [7–12]. These driving modes control the motion of the micro-nanorobots through external equipment such as the light, magnetic, and electric fields and other physical environments. Besides, through some redox reactions, bubbles are realized to promote the motion of a micro-nanorobot. However, both physical field driving and chemical driving have encountered some deficiencies related to economic and biocompatibility. Among which, the chemical drive was usually fueled by hydrogen peroxide, which had poor biocompatibility. Physical field driving usually needs to build an external environment platform, which was complicated and expensive. To improve the dynamics and the biocompatibility of micro-nanorobots, in this paper, we propose a micro-nanorobot with high biocompatibility by using glucose as fuel in human body fluids [13, 14].

Glucose fuel cells come in three types, including enzyme-catalyzed fuel cells, biocell-catalyzed fuel cells, and inorganic catalyzed fuel cells [15, 16]. An enzyme-catalyzed fuel cell has the advantage of high energy density. However, the degradation of the enzyme affected its service life, and the biological tissue used for natural cell catalysis was not suitable for natural implantation as well. Therefore, the principle of an inorganic catalytic fuel cell will need to be adopted.

## 2. Materials and Methods

### 2.1. Drive Mechanism

**Figure 1:**
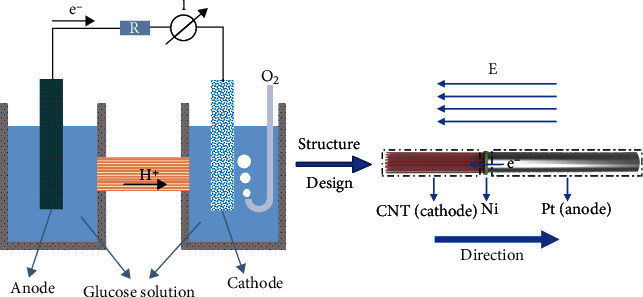
Drive mechanism of a Pt/CNT micro-nanorobot.

Through the analysis of the mechanism of a glucose galvanic cell, as shown in Figure 1, when the cell works, the cathode of the glucose electrolytic cell is reduced, and the anode is oxidized. Electrons in the cell flow from the anode to the cathode. This creates an electric field in the external environment to drive a current. In this paper, we compared the electric potential of different materials using the redox reaction.  Table 1 compares the open-loop potential of different cathode materials, in which the higher the potential, the higher the reduction efficiency.  Table 2 compares the open-loop potential of the anode materials, where the lower the potential, the higher the oxidation efficiency [17]. And platinum and activated carbon (carbon nanotube (CNT)) have high catalytic efficiency as the electrodes pair. CNT has excellent reducibility and could catalyze the decomposition of glucose, as well as has excellent electrical properties; so platinum and CNT were selected as the electrode materials [18]. Based on the principle of self-electrophoresis of micro-nanorobots, the asymmetric linear structure was designed. The driving mechanism of the Pt/CNT micro-nanorobot was obtained according to the catalytic decomposition principle of a glucose fuel cell. The oxidation reaction of glucose takes priority on the anode (Pt), while the reduction reaction is focused on the cathode (CNT). The electrons are distributed asymmetrically at both ends of the nanorods by the electric field created near the nanorods. Due to the negative charge on its metal surface, the robot moves in its self-generated electric field. The specific redox reaction principle is shown in equations (1), (2), and (3).

(1) $$\begin{array}{c} {\text{C}}_{6}{\text{H}}_{12}{\text{O}}_{6}+{\text{H}}_{2}\text{O}⟶{\text{C}}_{6}{\text{H}}_{12}{\text{O}}_{7}+2{\text{H}}^{+}+2{\text{e}}^{-} \end{array}$$

(2) $$\begin{array}{c} {\text{O}}_{2}+4{\text{H}}^{+}+4{\text{e}}^{-}⟶2{\text{H}}_{2}\text{O} \end{array}$$

(3) $$\begin{array}{c} {\text{C}}_{6}{\text{H}}_{12}{\text{O}}_{6}+\frac{1}{2}{\text{O}}_{2}⟶{\text{C}}_{6}{\text{H}}_{12}{\text{O}}_{7} \end{array}$$

### 2.2. Experimental Design

The principle of template electrochemical deposition was adopted to fabricate the structure of the anode material of the micro-nanorobot, and the three-electrode electrochemical deposition system was applied. The anode aluminum oxide (AAO) was applied as the template, and the cathode gold (Au) film was deposited by magnetron sputtering. Metal nanowires were deposited by the direct current electrodeposition method, and platinum nanowires were deposited by chloroplatinic acid (H_2_PtCl_6_) and boric acid (H_3_BO_3_). Ni thin films were deposited by nickel sulfate (NiSO_4_) and boric acid (H_3_BO_3_). Ferrous sulfate (FeSO_4_·7H_2_O) and boric acid (H_3_BO_3_) were applied for synthesizing the iron (Fe) nanoparticles. The growth of the cathode carbon nanotubes (CNT) was carried out by chemical vapor deposition. Ethylene (C_2_H_4_) was applied as the carbon source gas, with hydrogen as the reducing gas and argon as the protective gas. The deposition temperature was 750°C. The template was dissolved by sodium hydroxide (NaOH). The morphology of the micro-nanorobot was characterized by both SEM and TEM, and the element content of the micro-nanorobot was analyzed by EDX.

#### 2.2.1. Magnetron Sputtering for Cathode Gold Film

**Figure 2:**
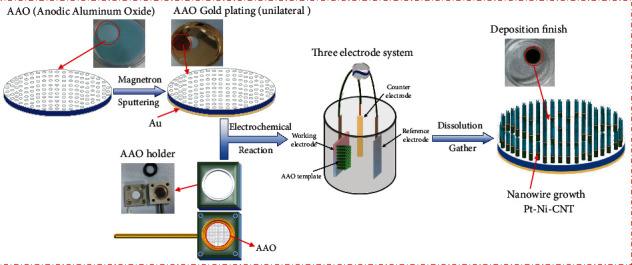
Technological process.

The AAO template was used for the working electrode of the three-electrode system. When using the AAO template for the electrode position, because the AAO template itself is not conductive, it is necessary to precover one side of it with a conductive metal layer. In this experiment, a magnetron plating instrument was used to sputter the cathode gold film on the AAO template to form such a conducting layer. To ensure good electrical conductivity and the gold film having covered the holes of the AAO template, the cathode gold film was required to be thicker than 50 nm. As shown in Figure 2, the template had been sputtered and been placed in the template support. The gold film was in contact with the copper electrode as the working electrode of the three electrodes. The counter electrode was selected as the Pt electrode because of its stability in the working environment. The reference electrode was the common calomel electrode. In the preparation technology, all the three factors of the solution concentration, the deposition time, and the deposition potential will influence the size and morphology of the micro-nanorobot. This paper designed the preparation experiment for a micro-nanorobot according to the above three factors.

#### 2.2.2. Technical Parameters of the Pt Nanowires, Ni Films, and Fe Particle Preparations

**Figure 3:**
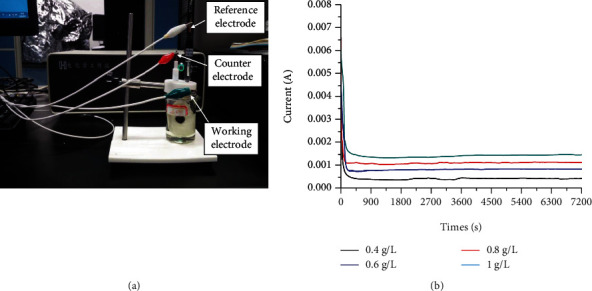
(a) Three-electrode electrochemical working experiment platform and (b) current diagram of Pt nanowire deposition at different concentrations.

As the anode of the glucose galvanic cell system, there are specific requirements on the Pt material in terms of size and morphology. The Pt nanowires were deposited with chloroplatinic acid (H_2_PtCl_6_) solution as the sedimentation effusion, and boric acid (H_3_BO_3_) and ascorbic acid (vitamin C) were added at the same time. Boric acid can stabilize the pH value of the deposition solution and prevent potential rapid deposition reaction from blocking the holes of the AAO template, which would otherwise lead to the nanowire deposition difficulty. The ascorbic acid can prevent certain cations from being oxidized to high-valence ions. The pH test was carried out on the sedimentation liquid, and a small amount of hydrochloric acid was added to adjust the pH value to about 3. Both the length and the diameter of the nanowires were changed by adjusting the concentration of the leading salt, the deposition potential, and the deposition time. This was a constant voltage deposition. To get the length of 3 *μ*m Pt nanowires, the electrochemical deposition experiments were carried out at different main salt concentrations, including 0.4 g/l, 0.6 g/l, 0.8 g/l, and 1 g/l of H_2_PtCl_6_ solutions. It was concluded that when the main salt concentration of the chloroplatinic acid was low, the Pt grains deposited in the AAO template holes were slow. Therefore, when the concentration of chloroplatinic acid was low, say, <0.4 g/l, the morphology of the deposited Pt was tubular. With increase of the concentration of the chloroplatinic acid, Pt continues to increase and the cavity in the middle of the tubulars became smaller. When the concentration of the chloroplatinic acid reached 1 g/l, the Pt grains deposited showed linear morphology rather than tubular. At the same time, the structures of Pt nanowires were also observed under different deposition potentials, including -0.1 V, -0.2 V, -0.3 V, -0.4 V, and -0.5 V, and different deposition time durations of 1 h, 2 h, 3 h, and 4 h. According to the SEM characterization results, the optimal experimental parameters of the obtained nanowire structure and size, including the optimal solution ratio, deposition potential, and deposition time, were obtained. The optimal main salt concentration of H_2_PtCl_6_ was 1 g/l, and the direct voltage was -0.3 V. The deposition current obtained is shown in Figure 3(b). Due to the different concentrations of H_2_PtCl_6_, the magnitude of the current was different, and it mainly showed that as the concentration became higher, the current also increased. The current density determined the result of deposition; as can be seen from the figure, the current density was the highest, i.e., about 1.5 × 10^−4^ A, when the concentration of the H_2_PtCl_6_ solution was 1 g/l. The Pt grains grew better in this environment.

Due to the poor interfacial wettability between Pt and CNT, it was not easy to form good connections in a whole. As a catalyst for the growth of CNT, the binding force between Ni and CNT is relatively good, and the heterogeneous structure between Ni and Pt is very stable as well. Therefore, a 5 nm thick Ni film was added first to act as the interconnection layer between Pt and CNT, and the overall structure became more stable. The Ni thin films were also deposited in the same way by the electrochemical method, the deposition liquid was 0.8 g/l NiSO_4_ and 30 g/l H_3_BO_3_, and the deposition voltage was -0.8 V. The ascorbic acid was added at the same time, and then, the pH value was tested. A small amount of sulfuric acid was added for pH adjustment so that the pH value of the solution kept at about 3.5. The thickness of the Ni films was also controlled by different deposition times. The experiments were carried out for 0.5 h, 1 h, and 2 h, respectively, to study the time influence on the Ni film deposition. Of course, the nickel film was over thicker when the deposition time was extended. However, the Ni film was only used as a connecting layer and only a thin film of about 5 nm was needed, for which only 1 min was fine to deposit the film.

As a catalyst for the growth of CNT, Fe particles affected the structure and performance of the CNT obtained. If the Fe particles grew into Fe nanowires, the growth environment of CNT was restricted. Therefore, experiments were also carried out to study the growth of the Fe particles. The precipitated liquid was configured with 120 g/l FeSO_4_·7H_2_O, 45 g/l H_3_BO_3_, and 1 g/l vitamin C. The deposition voltage was -1.5 V and the deposition time was 1 min.

#### 2.2.3. CNT Growth by Chemical Vapor Deposition

**Figure 4:**
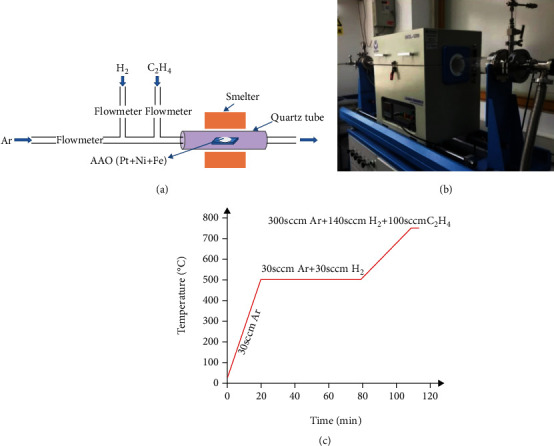
CNT growth process: (a) schematic diagram of chemical vapor deposition; (b) 1200°C tube furnace; (c) CVD time-temperature curve.

After the anode Pt of the micro-nanorobot had been prepared, we chose the chemical vapor deposition to prepare the cathode material, i.e., the CNT. The CNT was grown at the temperature of 1200°C in a sliding tube furnace to keep the high temperature, as shown in Figure 4(b). Figure 4(a) shows the schematic diagram of the CNT growth by chemical vapor deposition. The AAO template with Pt nanowires, Ni film, and Fe particles was placed in the quartz boat and then the whole module was put into the tube furnace. The thermal resistance wire of the tubular furnace should be placed under the quartz boat. The AAO template was set in the quartz boat, and the circulation of air was prevented. To obtain the required size and the morphology of the CNT, the experiment process was carefully designed with consideration of the carbon source gas flow rate and the deposition time.

Firstly, we closed the quartz tube and then turned on the vacuum pump to extract the air in the tube to make it vacuum. Then, according to the temperature curve as shown in Figure 4(c), argon (Ar) gas with flow rate of about 30 sccm was poured in to form a standard atmospheric pressure. The quartz tube was heated to 500°C within 20 min, and then, hydrogen gas (H_2_) of 30 sccm was injected. The temperature was maintained at 500°C for 1 h to ensure the reduction of Fe nanoparticles. Ethylene (C_2_H_4_) was used as the carbon source gas and poured into the quartz tube for reductive reaction at 750°C for 3 min. When the deposition was finished, the heating was stopped and the C_2_H_4_ and H_2_ gases were turned off, and the Ar flow rate was adjusted to 30 sccm to discharge the residual C_2_H_4_ and H_2_ through the tube. Ar was always kept in the tubular furnace as a protective gas. It lasted for 10 min to make sure the AAO template with carbon nanotubes deposited in a vacuum state and get cooled down to room temperature.

Three groups of experiments were carried out with different gas flow ratios of 100 sccm, 200 sccm, and 300 sccm, respectively. The results showed that if C_2_H_4_ flowed with a higher rate, the carbon nanotubes obtained would wind around each other, resulting in serious accumulation of carbon nanotubes and profound carbonization of graphite, and the most appropriate C_2_H_4_ flow rate was found to be100 sccm. In the growth process, the growth time of the carbon nanotubes was also considered with adjusting the deposition time for 1 min, 3 min, and 5 min, respectively. According to the results, if the deposition time was too short, the carbon nanotubes would be interwoven in a network. If the time was too long, the carbon nanotubes began to pile up and form the shape of carbon rods with large diameters. Only when the deposition time was about 3 min, the carbon nanotubes prepared were fibrous and ideal.

### 2.3. Materials

Anodic aluminum oxide (AAO) (diameter: 13 mm; different bore diameter: 50/70/90/200/300 nm) was purchased from Shangmu Tech, China. Chloroplatinic acid (H_2_PtCl_6_) (1 g/l), boric acid (H_3_BO_3_) (99.8 wt%), nickel sulfate (NiSO_4_) (99.9 wt%), ferrous sulfate (FeSO_4_·7H_2_O), and sodium hydroxide (NaOH) (97 wt %) were obtained from Aladdin, China.

Scanning electron microscopy (SEM) photos were taken by a ZEISS Merlin. Transmission electron microscopy (TEM) and energy-dispersive X-ray spectroscopy (EDX) images were taken by an FEI Talos. The electrochemical data was obtained from Metrohm Autolab (CHI600E, CH Instruments, U.S.A.).

An optical microscope (ZEISS Axiovert 40C, Germany) coupled with a ×40 objective and a high-speed camera (POINT GREY FL3-U3-13S2C-CS) along with visual software was used to record the motion videos (1328 × 1048 pixel, 120 fps).

## 3. Results

### 3.1. Morphology Observation and Component Analysis

**Figure 5:**
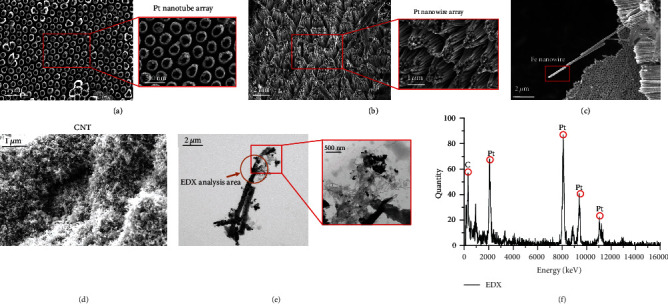
SEM images of (a) Pt nanotube array (diameter: 200 nm); (b) Pt nanowire array (diameter: 90 nm); (c) Fe nanowire (diameter: 90 nm); (d) CNT; (e) TEM image of the Pt/CNT micro-nanorobot; (f) EDX analysis.

The micro-nanorobot was prepared according to the above process. As shown in Figure 5, the SEM and TEM were applied to observe the structure of the Pt/CNT micro-nanorobot. Figure 5(a) shows the Pt nanotube array. This array was prepared by the electrodeposition process by a 200 nm AAO template working electrode. When the aperture of the AAO template was 90 nm, the Pt was a linear structure, as shown in Figure 5(b). Figure 5(c) shows the Fe nanowires; because the Fe deposition time was 2 min, the Fe particles became the Fe nanowires. Figure 5(d) shows the CNT prepared by the chemical vapor deposition (CVD) with the ethylene flow rate of 100 sccm and the deposition time of 3 min. The length of the Pt/CNT micro-nanorobot is about 8 *μ*m, and the tail is carbon nanotubes, as was shown in Figure 5(e). The morphology of the carbon nanotubes is mainly in the agglomeration shape, and the diameter of the carbon nanotubes is about 34 nm. To ensure the elemental composition of the micro-nanorobot, energy-dispersive X-ray spectroscopy (EDX) was employed to analyze the component of the Pt/CNT micro-nanorobot. It has confirmed the Pt/CNT element concentration distribution, as shown in Figure 5(f) that the content of Pt was higher than CNT because the CNT grew in the tail and was tubular. However, the Pt is in nanowires with body length longer than CNT, so the Pt content is higher than that of the carbon. The results were consistent with the prediction of the design of the structure.

### 3.2. The Motion Experiment of the Micro-Nanorobot

**Figure 6:**
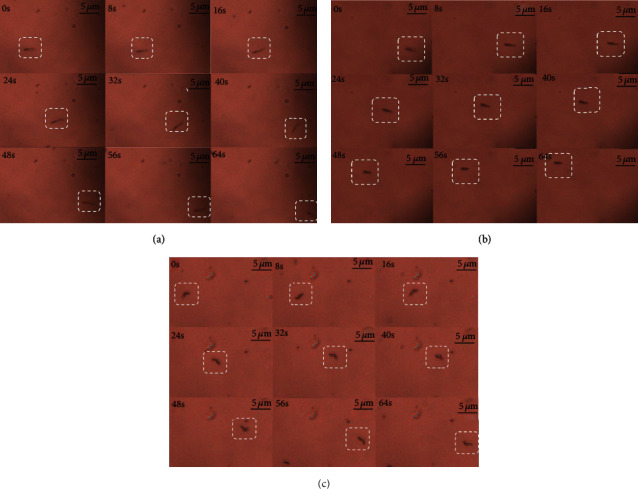
The motion state of the micro-nanorobot with different lengths: (a) 4.96 *μ*m; (b) 4.13 *μ*m; (c) 3.32 *μ*m.

**Figure 7:**
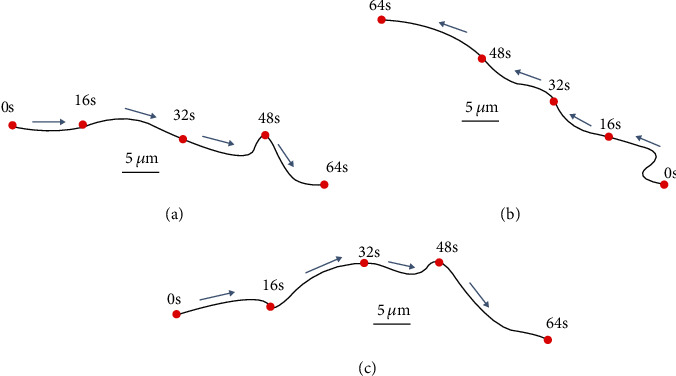
The motion trajectories of micro-nanorobots of different lengths: (a) 4.96 *μ*m; (b) 4.13 *μ*m; (c) 3.32 *μ*m.

The Pt/CNT micro-nanorobot was placed in a low concentration of blood glucose environment (30 wt%), and its motion state was characterized by a microscope.  Figure 6 shows the Pt/CNT micro-nanorobot with different body lengths. The solution containing the Pt/CNT micro-nanorobot was absorbed with a dropper and placed on a slide. The microscope was connected with a high-speed CCD with a frame rate of image capture of 120 fps. This experiment recorded the movements of the Pt/CNT micro-nanorobots with body lengths of 4.96 *μ*m (Figure 6(a)), 4.13 *μ*m (Figure 6(b)), and 3.32 *μ*m (Figure 6(c)), respectively. The motion videos were recorded and extracted by a video tracking software (ImageJ). The change of motion state of the Pt/CNT micro-nanorobot in 64 s was extracted as shown in Figure 7.

It could be seen from the movement trajectory of the Pt/CNT micro-nanorobot that, in the local motion range of the micro-nanorobot, it moved along the Pt side; however, because of its small size, it was obviously affected by the Brownian force and the trajectory of the micro-nanorobot was a curve. According to the collected pictures and time-prints and the kinematic calculation model of the Pt/CNT micro-nanorobot, the movement speed of the micro-nanorobot could be estimated. The body length of the micro-nanorobot was 3.32 *μ*m, whose length of pixels in the image was about 0.83. The micro-nanorobot moved the pixel distance from the first frame to the last frame which was about 7.38, so the actual movement distance was more than the body length of the micro-nanorobot, i.e., about 8.9 times. The actual movement distance was 29.5 *μ*m. The motion state of the micro-nanorobot was approximate to the uniform motion. According to equation (4), it was analyzed that the micro-nanorobot movement speed was about 0.46 *μ*m/s. Similarly, the movement speed of the micro-nanorobot, whose length was 4.13 *μ*m and 4.96 *μ*m, could be calculated.

According to the kinematic calculation model of the Pt/CNT micro-nanorobot, the Stokes viscous force of the cylinder is shown in equation (5). The micro-nanorobot was exposed to the concentration of 30% glucose solution, whose dynamic viscosity was 2.23 × 10^−3^ Pa · s. The diameter of the anodic aluminum oxide template (AAO) was about 90 nm. The length of the micro-nanorobot is 3.32 *μ*m, 4.13 *μ*m, and 4.96 *μ*m, whose movement speed was about 0.46 *μ*m/s, 0.37 *μ*m/s, and 0.34 *μ*m/s, respectively, and then the calculated size of the viscous force was 1.02 × 10^−14^ N, 0.92 × 10^−14^ N, and 0.87 × 10^−14^ N by equation (5). According to the video sequence, it could be observed that the motion of the micro-nanorobot was basically at a constant speed, and the acceleration of the micro-nanorobot was tiny. Because the result of the weight multiplied with the acceleration was very small, the value of the driving force should be equal to the viscous force. It could be seen then that the driving force of the Pt/CNT micro-nanorobot was the same as the viscous force when its body length was between 3 to 5 *μ*m, and the order of magnitude of the driving force was about 10^−14^ N. The results are shown in Table 3.

(4) $$\begin{array}{c} V=\frac{L}{t}, \end{array}$$

(5) $$\begin{array}{c} F_{d}=\frac{2\pi \mu L}{\ln\left(2L/r\right)-0.72}V_{s}. \end{array}$$

## 4. Discussion

This paper studied the micro-nanorobot that used glucose as fuel and realized the self-actuation of micro-nanorobots in glucose, which has vital research significance. Based on the driving principle of self-electrophoresis and the catalytic decomposition principle of a glucose fuel cell, this paper designed the structure of the Pt/CNT micro-nanorobot. According to the system, the processing of the Pt/CNT micro-nanorobot was carried out by selecting the combination of electrochemical deposition and chemical vapor deposition. SEM and TEM were employed to characterize the Pt/CNT micro-nanorobot. The issue of accurate trajectory control for a micro-nanorobot was not involved in the experiment. A more controllable Pt/CNT micro-nanorobot with biological compatibility will need further studies including the research of the Pt-CNT connective structure, the effect of the thickness of the Ni membrane through improved electrochemical deposition, and external magnetic driving devices to control the direction of motion of the micro-nanorobot. The Pt/CNT micro-nanorobot prepared here would help to achieve potential medical applications on target therapy, biosensor, drug carrier, and others.

## Figures and Tables

**Table 1:** Cathodic catalyzed materials' opening loop potential [17].

Materials	Open-loop potential (mV)
C	877
Au	710
Ag	590

**Table 2:** Anodic catalytic materials' opening loop potential [17].

Materials	Open-loop potential (mV)
Pt	180
Rh	120
Ir	90

**Table 3:** The driving force and driving speed of the Pt/CNT micro-nanorobot of different lengths in a glucose solution with a concentration of 30%.

Micro-nanorobot length (*μ*m)	Driving force (N)	Speed (*μ*m/s)
3.32	1.02 × 10^−14^	0.46
4.13	0.92 × 10^−14^	0.37
4.96	0.87 × 10^−14^	0.34

Compared with the micro-nanorobot using hydrogen peroxide (H_2_O_2_) as fuel, the Pt/CNT micro-nanorobot had smaller driving force because the catalytic decomposition efficiency of glucose was lower than that of hydrogen peroxide.

## Data Availability

The data used to support the findings of this study are included within the article.
